# Molecular epidemiology of rodent-borne Leptospira spp. in Sri Lanka: identification of novel sequence types and previously unrecognized reservoir animals

**DOI:** 10.1099/jmm.0.002133

**Published:** 2026-03-06

**Authors:** Nipun Rathnayake, Kyosuke Takabe, Devinda Muthusinghe, Rydhnieya Vijeyakumaran, Pavani Senarathne, Nilanthi Dissanayake, Shuzo Urata, Kumiko Yoshimatsu, Yukihiro Akeda, Chandika Gamage, Nobuo Koizumi

**Affiliations:** 1 Department of Microbiology, Faculty of Medicine, University of Peradeniya, Peradeniya, Sri Lanka; 2 Department of Bacteriology I, National Institute of Infectious Diseases, Japan Institute for Health Security, Tokyo, Japan; 3 National Research Center for the Control and Prevention of Infectious Diseases, Nagasaki University, Nagasaki, Japan; 4 Graduate School of Infectious Diseases, Hokkaido University, Sapporo, Japan; 5 Laboratory of Animal Experimentations, Institute for Genetic Medicine, Hokkaido University, Sapporo, Japan; 6 Department of Pathobiological Sciences, School of Veterinary Medicine, Louisiana State University, Baton Rouge, LA, USA

**Keywords:** *Leptospira *spp., leptospirosis, MLST, rodent, Sri Lanka

## Abstract

**Introduction.** Leptospirosis is an important zoonotic disease globally, which is most prevalent in tropical regions. This disease is endemic in Sri Lanka, where the complex ecology of *Leptospira* spp., reservoir animals and environmental and occupational factors has resulted in a public health problem.

**Gap Statement.** Although genomic analysis of *Leptospira* isolates has recently revealed the diversity of *Leptospira* spp. in Sri Lanka, the genetic relationship between human patients and reservoir animals remains unclear.

**Aim.** This study investigated the genetic diversity of *Leptospira* spp. circulating in rodent populations in three districts of Sri Lanka: Kurunegala, Anuradhapura and Badulla.

**Methodology.** *Leptospira* DNA was detected from rodent kidney tissue samples by real-time PCR, from which positive samples were subjected to *flaB* sequencing and multilocus sequencing typing (MLST).

**Results.** Pathogenic *Leptospira* DNA was detected by real-time PCR in 33 of 257 kidney tissue samples (12.8%) from 4 rodent species: *Bandicota bengalensis*, *Mus booduga*, *Rattus rattus* and *Vandeleuria* sp. MLST and partial *flaB* sequencing of real-time PCR-positive samples identified *Leptospira borgpetersenii*, *Leptospira interrogans*, *Leptospira kirschneri* and *Leptospira licerasiae* in the rodent population. Five sequence types (STs), including two novel STs, ST389 and ST392, were identified. The novel STs of *L. interrogans* and *L. kirschneri* were genetically distinct from other STs detected in Sri Lanka. *R. rattus* and *M. booduga* were newly identified as the source of *L. interrogans* ST49 and of *L. borgpetersenii* ST144 and *L. licerasiae* infections in humans, respectively.

**Conclusion.** This study identified the genetic diversity of *Leptospira* spp. in rodent populations and reservoir animals for human infections in Sri Lanka.

## Introduction

Leptospirosis is one of the most prevalent zoonoses and is caused by pathogenic spirochetes of the genus *Leptospira*. It is estimated that approximately one million leptospirosis cases and 58,900 associated deaths occur annually worldwide, of which more than 70% occur in the tropics [1]. However, leptospirosis is underdiagnosed in many tropical regions, mainly due to its diverse, nonspecific clinical manifestations that are similar to those of many other infectious diseases such as dengue fever, and limited or absent capacity of laboratories to diagnose it [2,5]. Pathogenic *Leptospira* spp. colonize the proximal renal tubules and are excreted in the urine of maintenance host animals [6,7]. Humans are infected with *Leptospira* spp. through damaged skin or mucous membranes mainly by exposure to water or soil contaminated with the urine of maintenance hosts [6,8]. Although rodents are important maintenance hosts of *Leptospira* spp., domestic animals, such as dogs, cattle and pigs, and wildlife species can also be reservoirs of certain *Leptospira* strains (serovars) [6,8]. *Leptospira* spp. comprise pathogenic and saprophytic species, which are divided into four subclades: P1 and P2 for pathogenic species and S1 and S2 for saprophytic ones [9]. In the pathogenic subclade, P1 contains pathogenic species responsible for human and animal infections. Although P2 subclade species are considered to have intermediate pathogenicity and are less virulent than P1 pathogens, human infection with P2 subclade *Leptospira* spp. has been reported in various locations globally [10,15].

Sri Lanka is recognized as a hotspot for leptospirosis, with an estimated annual incidence of 52.1 cases per 100,000 population and ~730 deaths [16]. Leptospirosis cases have been reported in almost all regions of the country, with a high incidence in its wet zones [17]. Within the last decade, the highest number of clinically suspected cases, 9,630 cases and 203 deaths, was reported in 2023 [18], which was the highest incidence since the 2008 outbreak [19,20]. This increase may be related to several factors, including an increase in agricultural activities due to the country’s economic crisis, increased rainfall, higher detection in advances in laboratory facilities and improved surveillance [21]. The prevalence of leptospirosis in Sri Lanka is influenced by environmental, weather-related, occupational and behavioural factors. Among these, agricultural exposure, particularly rice farming, is the most important [22,23]. The widespread practice of rice farming involves flooded or muddy fields, especially during the rainy season, creating optimal conditions for *Leptospira* bacteria. Farmers working barefoot in these paddy fields are particularly vulnerable to exposure [22]. Leptospirosis is known as rat fever in this country [24], and *Leptospira* spp. have been detected in rodents and shrews, such as black rats (*Rattus rattus*), lesser bandicoot rats (*Bandicota bengalensis*), Indian bandicoot rats (*Bandicota indica*), little Indian field mice (*Mus booduga*) and Asian house shrews (*Suncus murinus*) [25,26]. In addition to rodents, *Leptospira* spp. have been detected in companion and domestic animals such as dogs, cattle, buffaloes and elephants [25,27,29].

Although many *Leptospira* isolates were obtained from humans, rodents, shrews and dogs in the 1960s and early 1970s [30,31], recent studies have again started to successfully isolate *Leptospira* spp. in Sri Lanka [26,32, 33]. In 2020, multilocus sequencing typing (MLST) revealed 15 sequence types (STs), including 6 new STs from the isolates of 3 *Leptospira* species, *Leptospira borgpetersenii*, *Leptospira interrogans* and *Leptospira kirschneri*, from human patients and black rats in Sri Lanka [26] In 2022, six STs, including four new STs, from the above three species were identified in rodent populations [25]. Whole-genome sequencing (WGS) of 25 *Leptospira* isolates from human patients, including *L. borgpetersenii*, *L. interrogans*, *L. kirschneri* and *Leptospira weilii*, identified 15 clonal groups, including 12 groups that had not previously been reported [33]. A WGS study also showed that *L. kirschneri* isolates from febrile patients formed a distinct phylogenetic cluster from other *L. kirschneri* strains [34]. In addition to these four *Leptospira* species, *Leptospira kmetyii* and *Leptospira licerasiae* DNA has been detected in humans and other animals [35,36]. The diversity of *Leptospira* spp. highlights the importance of environmental and animal reservoirs in disease transmission in Sri Lanka. However, although black rats and Asian house shrews are the source of human infection for *L. borgpetersenii* ST144 [26,32, 33], the reservoir animals for other *Leptospira* genotypes remain unknown.

In this study, we detected *Leptospira* DNA in rodent kidney tissue samples by real-time PCR targeting *lipL32* and *rrs* in three districts of Sri Lanka: Kurunegala, Anuradhapura and Badulla. The real-time PCR-positive samples were subjected to MLST using seven housekeeping genes and *flaB* sequencing to identify *Leptospira* species and genotypes (STs).

## Methods

### Sample collection from the rodents

**Fig. 1.**
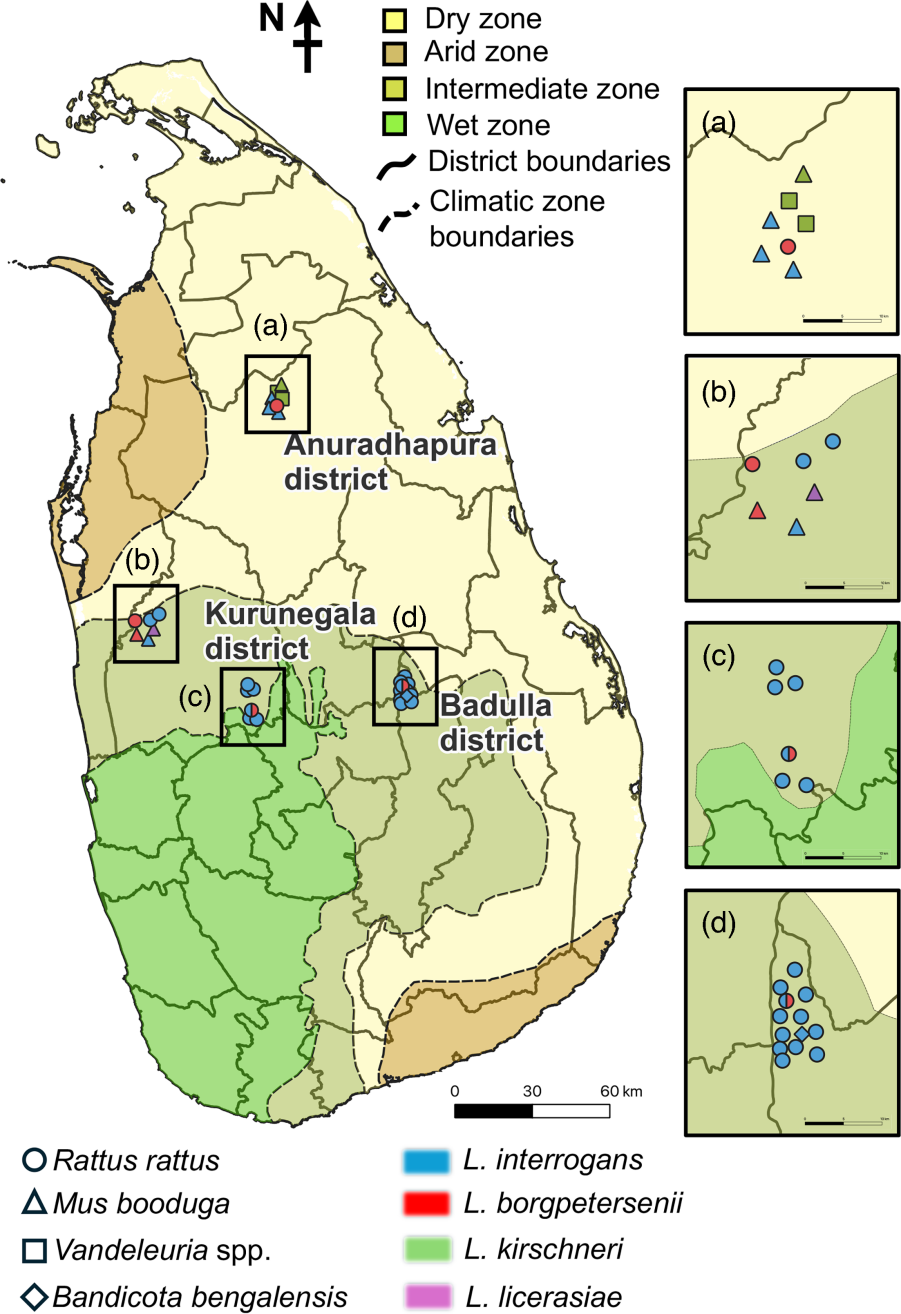
A map of Sri Lanka showing the capture sites of *Leptospira*-positive rodents in relation to climatic zones. Geographical distribution of *Leptospira*-positive rodents captured in three districts of Sri Lanka plotted onto the country’s climatic zones. Colour shading indicates the climatic zones (dry, arid, intermediate and wet). District boundaries and climatic zone boundaries are shown by solid and dashed lines, respectively. Insets (a–d) present enlarged views of the specific trapping locations within each district. Symbols denote rodent species (*R. rattus*, *M. booduga*, *Vandeleuria* spp. and *B. bengalensis*), and colours indicate *Leptospira* species detected (*L. interrogans*, *L. borgpetersenii*, *L. kirschneri* and *L. licerasiae*).

A total of 257 rodents were captured using live traps in paddy fields, agricultural areas and nearby houses from three districts of Sri Lanka, Kurunegala (*n*=79), Anuradhapura (*n*=61) and Badulla (*n*=117), between 2016 and 2024 (Fig. 1). The rodents were humanely euthanized using isoflurane inhalation, following the guidelines of the American Veterinary Medical Association, and kidney tissues were collected and stored at −25 °C prior to DNA extraction. Ethical approval for this study was obtained from the ethical review committee of the Faculty of Veterinary Medicine and Animal Sciences, University of Peradeniya (ethical approval no.: VERC-22-03).

### Detection of *Leptospira* DNA from rodent kidney tissue

DNA was extracted from ~25 mg of rodent kidney tissue cortex using the DNeasy Blood and Tissue Kit (Qiagen, Hilden, Germany). The extracted DNA was then subjected to real-time PCR for *lipL32* and *rrs*. For *lipL32* detection, the sequences of the primers were the same as previously described [37], while the probe contained the ZEN quencher: 5′-FAM-AAAGCCAGG/ZEN/ACAAGCGCCG-IBFQ-3′, and the concentrations of the primers and the probe used were 0.3 and 0.2 µM, respectively. For *rrs* detection, 0.4 µM of the forward primer F3C (5′-TCATTGGGCGTAAAGGGTGC-3′), 0.6 µM of the reverse primer B3C (5′-TCAGTTTTAGGCCAGCAAGTC-3′) and 0.25 µM of the double-quenched probe DQ16SP (5′-FAM-AGAGGCAAG/ZEN/TGGAATTCCAGGTG-IBFQ-3′) were used. The real-time PCR was performed with Thunderbird Probe qPCR Mix (Toyobo, Osaka, Japan) on a LightCycler 96 (Roche, Basel, Switzerland): after an initial denaturation step at 95 °C for 60 s, the reaction mixture was subjected to 40 cycles of denaturation at 95 °C for 10 s and amplification at 60 °C for 60 s.

### *flaB*-nested PCR

The real-time PCR-positive DNA samples were subjected to nested PCR targeting *flaB* for the P1 subclade *Leptospira* spp. [38] and the P2 subclade *Leptospira* spp. For P2 subclade *Leptospira flaB* amplification, the first PCR primer set L-I*flaB*-F1 (5′-CCGCTCTCTGAAGTTCAACGAG-3′) and L-I*flaB*-R1 (5′-GTTTGAGGACCGAATTCGGTTTT-3′) and the second PCR primer set L-I*flaB*-F2 (5′-GAGCWAGCTGTGGATAAAACC-3′) and L-I*flaB*-R2 (5′-CTAACATCGCCGTACCACTCTGCA-3′) were used. The reaction conditions were the same as previously described [38]. DNA sequencing of the amplicons was performed with the second primers using the BigDye Terminator v3.1 Cycle Sequencing Kit (Applied Biosystems, Foster City, CA, USA). The nucleotide sequences of *flaB* detected were deposited in a public database (DDBJ accession numbers LC869567–LC869580).

### MLST

MLST was applied to real-time PCR-positive DNA samples, and seven housekeeping genes, *glmU*, *pntA*, *sucA*, *tpiA*, *pfkB*, *mreA* and *caiB*, were amplified as previously described [39,40]. DNA sequencing of the amplicons was performed using the M13 primers as described above. The nucleotide sequences of the above genes have been deposited in a public database (DDBJ; accession numbers LC869459–LC869566). New allele sequences have also been deposited in the *Leptospira* PubMLST database (https://pubmlst.org/organisms/leptospira-spp). A minimum spanning tree (MST) based on the allelic profiles of MLST of *L. interrogans* and *L. kirschneri* determined in this study and strains deposited in the MLST database was constructed using GrapeTree [41].

### Identification of the rodent species

Rodent species were identified morphologically, and those from which leptospiral DNA was detected were further confirmed by sequencing of the mitochondrial cytochrome b (*Cytb*) gene. The *Cytb* gene was amplified using the primer set L14115 and H655A [42], and the amplicons were sequenced with the same primers. The resulting sequences were compared with those in public databases using the blast algorithm (https://blast.ncbi.nlm.nih.gov/Blast.cgi).

### Statistical analysis

The associations between the *Leptospira* carriage rate in rodents and the districts where rodents were captured were tested using a 2×3 chi-square test. A *P* value less than 0.05 was considered statistically significant.

## Results

### Detection of *Leptospira* DNA from rodent kidney tissues

Leptospiral DNA was detected in 20 and 27 of the 257 kidney tissue samples by real-time PCR for *lipL32* and *rrs*, respectively (Table 1). In total, 33 samples were positive by at least 1 assay. Both genes were detected in 14 samples, whereas *lipL32* alone or *rrs* alone was detected in 6 and 13 samples, respectively. These results indicate that the *rrs* assay detected a greater number of positives than the *lipL32* assay, suggesting higher analytical sensitivity in our rodent samples. Conversely, the six samples positive only by *lipL32* indicate that each assay may miss a subset of infections. The rodent species from which leptospiral DNA was detected were *R. rattus* (23 animals), *M. booduga* [7], *Vandeleuria* sp*.* [2] and *B. bengalensis* [1] (Tables 1 and 2). There was no significant difference in the carriage rate of *Leptospira* spp. among the districts where rodents were captured: Kurunegala (15.2%, 12 out of 79), Anuradhapura (11.5%, 7 out of 61) and Badulla (11.9%, 14 out of 117) [χ^2^ (2, *N*=257)=0.57, *P*=0.75] (Table 2).

**Table 1.** Results of real-time PCR, nested PCR, MLST and rodent species identification in this study^*^

Sample ID		Real-time PCR		*flaB*-nested PCR		MLST									Rodent species
		*lipL32*	*rrs*	P1 subclade	P2 subclade	*glmU*	*pntA*	*sucA*	*tpiA*	*pfkB*	*mreA*	*caiB*	ST	*Leptospira* species	
UP-RK-026	Kurunegala	P^†^	P	*L. interrogans*	−	5	1	1	1	3	2	7	ST49	*L. interrogans*	*R. rattus*
UP-RK-038	Kurunegala	−^‡^	P	−	−	−	−	M	−	10	−	−		*L. interrogans*	*R. rattus*
UP-RK-047	Kurunegala	−	P	−	−	M	−	3	−	**133**	−	−		*L. interrogans*	*R. rattus*
UP-RK-049	Kurunegala	−	P	−	−	−	3	3	−	9	−	−		*L. interrogans*	*R. rattus*
UP-RK-061	Kurunegala	P	P	−	−	77	86	M	78	M	77	56		*L. interrogans*	*R. rattus*
UP-RK-064	Kurunegala	P	P	*L. borgpetersenii/* *L. interrogans*	−	NT^||^	nt	nt	nt	nt	nt	nt			*R. rattus*
UP-RK-068	Kurunegala	−	P	−	−	3	−	−	−	−	5	−		*L. interrogans*	*M. booduga*
UP-RK-070	Kurunegala	−	P	−	*L. licerasiae*	nt	nt	nt	nt	nt	nt	nt			*M. booduga*
UP-RK-074	Kurunegala	−	P	−	−	−	−	−	−	9	5	−		*L. interrogans*	*R. rattus*
UP-RK-075	Kurunegala	P	P	−	−	24	27	30	34	67	27	28	ST144	*L. borgpetersenii*	*R. rattus*
UP-RK-076	Kurunegala	P	P	−	−	24	27	30	34	67	27	28	ST144	*L. borgpetersenii*	*M. booduga*
UP-RK-077	Kurunegala	P	−	−	−	−	−	−	−	9	−	−		*L. interrogans*	*R. rattus*
UP-RA-088	Anuradhapura	−	P	−	−	−	−	3	−	10	5	−		*L. interrogans*	*M. booduga*
UP-RA-089	Anuradhapura	−	P	−	−	−	−	−	−	M	−	−		*L. interrogans*	*M. booduga*
UP-RA-090	Anuradhapura	P	P	*L. borgpetersenii*	−	24	28	30	35	37	26	73	ST323	*L. borgpetersenii*	*R. rattus*
UP-RA-126	Anuradhapura	P	P	*L. kirschneri*	−	**93^¶^**	**102**	15	**94**	**130**	**89**	**85**	**ST389**	*L. kirschneri*	*Vandeleuria* sp.
UP-RA-127	Anuradhapura	P	P	*L. kirschneri*	−	**93**	**102**	15	**94**	**130**	**89**	**85**	**ST389**	*L. kirschneri*	*Vandeleuria* sp.
UP-RA-128	Anuradhapura	P	P	*L. kirschneri*	−	**93**	**102**	15	**94**	**130**	**89**	**85**	**ST389**	*L. kirschneri*	*M. booduga*
UP-RA-130	Anuradhapura	P	−	−	−	−	−	3	−	9	M	−		*L. interrogans*	*M. booduga*
UP-RB-156	Badulla	P	−	*L. interrogans*	−	−	−	−	−	−	5	−		*L. interrogans*	*R. rattus*
UP-RB-157	Badulla	−	P	−	−	−	−	−	−	−	−	−			*R. rattus*
UP-RB-178	Badulla	P	−	−	−	−	−	−	−	68	2	−		*L. interrogans*	*R. rattus*
UP-RB-181	Badulla	−	P	−	−	−	14	−	−	4	5	−		*L. interrogans*	*R. rattus*
UP-RB-182	Badulla	P	−	*L. interrogans*	−	8	14	2	17	10	5	6	**ST392**	*L. interrogans*	*R. rattus*
UP-RB-193	Badulla	−	P	−	−	−	−	2	17	26	−	M		*L. interrogans*	*B. bengalensis*
UP-RB-204	Badulla	P	P	*L. interrogans*	−	−	−	−	17	−	16	6		*L. interrogans*	*R. rattus*
UP-RB-210	Badulla	−	P	−	−	−	−	−	−	M	−	−		*L. interrogans*	*R. rattus*
UP-RB-212	Badulla	P	P	*L. interrogans*	−	8	14	2	17	M	M	6		*L. interrogans*	*R. rattus*
UP-RB-213	Badulla	−	P	−	−	−	−	−	−	M	5	8		*L. interrogans*	*R. rattus*
UP-RB-224	Badulla	P	P	*L. borgpetersenii*	−	**94**	−	3	35	**131**	5	**86**		*L. borgpetersenii*/ *L. interrogans*	*R. rattus*
UP-RB-225	Badulla	P	P	−	−	−	−	−	−	10	−	−		*L. interrogans*	*R. rattus*
UP-RB-233	Badulla	P	−	*L. interrogans*	−	−	−	−	−	**132**	4	7		*L. interrogans*	*R. rattus*
UP-RB-240	Badulla	P	P	*L. interrogans* (M^§^)	−	nt	nt	nt	nt	nt	nt	nt			*R. rattus*

*Only samples with at least one real-time PCR-positive result are listed.

†P: positive in PCR.

‡−: negative in PCR.

§M: mixed sequence.

||nt: not tested.

¶Novel alleles and STs identified in this study are shown in bold.

**Table 2.** *Leptospira* carriage rate among rodents captured in different climatic zones of Sri Lanka

Zone	District	Host species	No. of tested	No. of positive	% positive	Total positivity (no. of positive/no. of tested)
Dry	Anuradhapura	*B. bengalensis*	1	0	0.0	11.5 (7/61)
		*M. booduga*	45	4	8.9	
		*R. rattus*	10	1	10.0	
		*Tatera indica*	3	0	0.0	
		*Vandeleuria* sp.	2	2	100.0	
Intermediate	Kurunegala	*B. bengalensis*	28	0	0.0	15.2 (12/79)
		*M. booduga*	5	3	60.0	
		*R. rattus*	45	9	20.0	
		*S. murinus*	1	0	0.0	
	Badulla	*B. bengalensis*	1	1	100.0	12.0 (14/117)
		*M. booduga*	2	0	0.0	
		*R. rattus*	113	13	11.5	
		*Vandeleuria* sp.	1	0	0.0	

### Species identification and MLST for *Leptospira* DNA detected in rodent kidney tissues

*flaB* of the P1 and P2 subclades was detected in 13 and 1 of the 33 real-time PCR-positive samples, respectively (Table 1). Of the 33 samples positive by real-time PCR, 3 (2 with mixed profiles and 1 identified as a P2 species by *flaB* sequencing) were not subjected to MLST. Among the remaining 30 samples, at least 1 MLST gene was amplified in 29. The highest positivity was observed for *pfkB* (26 out of 30), while *pntA* showed the lowest (12 out of 30) (Table 1). There were 7 alleles in *glmU*, 7 in *pntA*, 5 in *sucA*, 6 in *tpiA*, 12 in *pfkB*, 8 in *mreA* and 8 in *caiB*. One new allele was found in *pntA*, *tpiA* and *mreA*, two in *glmU* and *caiB* and three in *pfkB* (Table 1). Combined with the sequences of *flaB* and seven housekeeping genes, four *Leptospira* spp. were identified in Sri Lankan rodents. Specifically, *L. interrogans*, *L. borgpetersenii*, *L. kirschneri* and *L. licerasiae* were identified in 24, 4, 3 and 1 rodents, respectively. Mixed infections, defined by either (i) the presence of double peaks in sequencing chromatograms or (ii) the detection of alleles originating from different *Leptospira* species (e.g. *L. interrogans* and *L. borgpetersenii*) across the 7 MLST loci or *flaB*, were observed in 12 of the 33 rodents (36.4%).

**Fig. 2.**
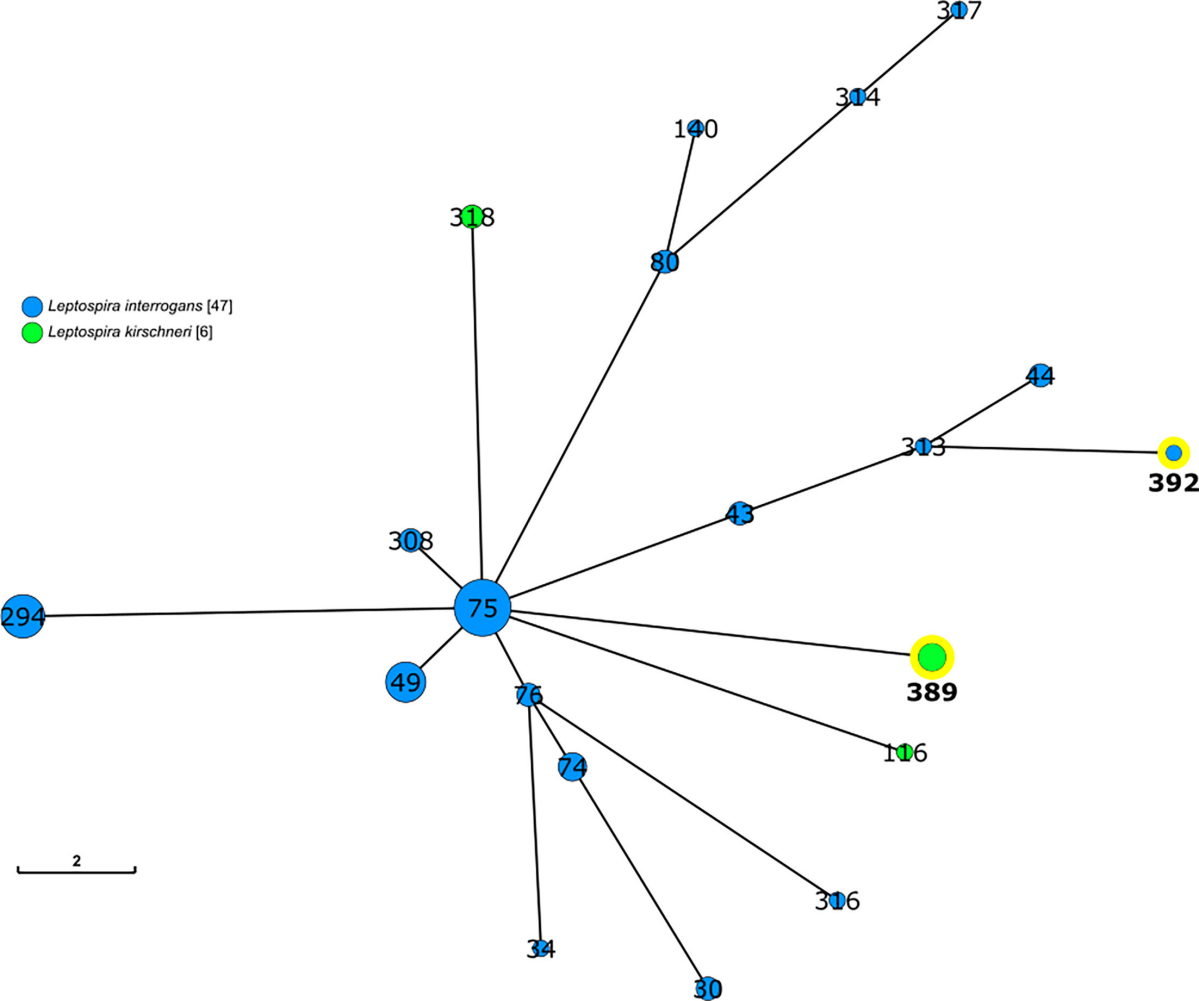
MST of *L. interrogans* and *L. kirschneri* strains in Sri Lanka. Each circle represents an ST, and the numbers indicate ST designations. Circle sizes are proportional to the number of strains assigned to each ST. Blue and green circles represent *L. interrogans* and *L. kirschneri*, respectively, and yellow-highlighted circles indicate the new STs identified in this study. The MST was constructed based on allelic profiles from the seven housekeeping genes of MLST scheme 1.

All seven loci were successfully determined in eight animals, including *L. borgpetersenii* ST144 in *R. rattus* and *M. booduga*, *L. borgpetersenii* ST323 in *R. rattus*, *L. interrogans* ST49 in *R. rattus*, a novel ST (ST392) of *L. interrogans* in *R. rattus* and a novel ST (ST389) of *L. kirschneri* in *Vandeleuria* sp. and *M. booduga* (Table 1). The novel *L. interrogans* ST392 consisted of the allele combination *glmU*: 8, *pntA*: 14, *sucA*: 2, *tpiA*: 17, *pfkB*: 10, *mreA*: 5 and *caiB*: 6. Five of these alleles were identical to those of ST57. The novel *L. kirschneri* ST389 consisted of six novel alleles (*glmU*: 93, *pntA*: 102, *tpiA*: 94, *pfkB*: 130, *mreA*: 89 and *caiB*: 85) and one previously known allele (*sucA*: 15) (Table 1). The MST indicated that *L. interrogans* ST392 and *L. kirschneri* ST389 were genetically distinct from the other Sri Lankan isolates (Figs 2, S1 and S2, available in the online Supplementary Material). The *flaB* sequence of *L. licerasiae* UP-RK-070 was identical to that detected in human blood in Sri Lanka (accession number LC752688 [36].

## Discussion

This study revealed that the kidney tissues of 12.8% (33 out of 257) of rodents in the Kurunegala, Anuradhapura and Badulla districts of Sri Lanka contained *Leptospira* spp. with high genetic diversity, including four *Leptospira* species and at least 12 genotypes, including 2 novel STs. Here, *Leptospira* spp. were detected in the black rat, *R. rattus*; the lesser bandicoot rat, *B. bengalensis*; and the little Indian field mouse, *M. booduga*, as reported previously [25,26]. However, to the best of our knowledge, this is the first study to identify the long-tailed mouse, *Vandeleuria* sp., as a carrier of *Leptospira*. The carriage rate in rodents was almost the same among the three regions. The areas where rodents were trapped in the Badulla and Kurunegala districts belong to the intermediate zone, while the Anuradhapura district belongs to the dry zone (Fig. 1, Table 2). Although the prevalence of rodents carrying *L. interrogans* in Southeast Asia is known to be higher in humid habitats than in dry ones [43], another study in Sri Lanka reported no difference in the carriage rate between rodents captured in the dry and intermediate zones [25,26], which may be due to the different genotypes of *L. interrogans* circulating in these areas.

MLST of *Leptospira* DNA in kidney tissue samples identified reservoir animals that are the source of infection for humans. *L. borgpetersenii* ST144 has been isolated from human patients, as well as black rats and shrews in Sri Lanka [26,32, 33], and this study identified the little Indian field mouse as the reservoir of this genotype in addition to black rat. *L. interrogans* ST49 has been isolated from humans in Sri Lanka (https://pubmlst.org/organisms/leptospira-spp), and this study demonstrated that black rats are the source of infection for this genotype in humans. In addition to the existing STs, this study also identified two novel STs: *L. interrogans* ST392 and *L. kirschneri* ST389 (Table 1, Figs 2, S1 and S2), although these STs have not been detected in human patients. The novel *L. interrogans* ST392 shared five of seven alleles with ST57, suggesting a close genetic relationship, whereas the new *L. kirschneri* ST389 comprised mostly novel alleles, representing a distinct lineage from previously identified Sri Lankan isolates (Table 1, Figs 2, S1 and S2). This suggests the presence of unique genetic variations in local *L. kirschneri* populations. Therefore, further studies are needed to investigate these novel genetic variations, particularly their role in zoonotic spread.

Human and animal infection with the P2 subclade of *Leptospira* spp. has been reported in various locations worldwide [10,15,44]. In Sri Lanka, *L. licerasiae* DNA has been detected in humans [35], dogs [35,36] and pigs (DDBJ accession number LC830693), and this study showed that the little Indian field mouse, *M. booduga*, is a potential source of *L. licerasiae* infection in humans. A wide variety of animals such as rats (*Rattus* spp. and *Proechimys* spp.), opossums (*Metachirus nudicaudatus*) and bats (*Uroderma magnirostrum*) are known to be reservoirs for *L. licerasiae* in the Peruvian Amazon [12], suggesting that other animals may be reservoirs for other genotypes of *L. licerasiae* in Sri Lanka. In addition, this study demonstrated the utility of real-time PCR for *rrs*, resulting in the identification of the mouse as a reservoir. It has been reported that some assays targeting *rrs* can detect DNA from other bacteria, especially when they are applied to urine samples [45,47]. The assay used in this study showed high sensitivity and specificity that are comparable with those of the assay targeting *lipL32* (Table 1), although no other genes were detected in 1 *rrs*-positive sample (Cq 36.57, the highest value in this study). Notably, the *rrs* assay identified more positive samples than *lipL32*, suggesting higher analytical sensitivity under our conditions. However, six samples were detected only by *lipL32*, indicating that both assays have inherent limitations. These discrepancies may be attributable to low DNA concentrations, stochastic amplification effects or sequence variation at primer and/or probe binding sites.

Mixed infections with the same or different species were observed in 36.4% (12 out of 33) of the animals from which *Leptospira* sequences were obtained. Mixed infections with *L. borgpetersenii* and *L. interrogans* in a single animal have been observed in different small mammals from around the world [48,49]. This common phenomenon poses a problem for PCR-based MLST: in the sample UP-RB-224, the sequences of *sucA* and *mreA* were derived from *L. interrogans*, while that of *tpiA* was from *L. borgpetersenii* (Table 1), indicating that each locus can be amplified from different strains infecting a single animal and that it is not possible to determine whether the sequences are from the same or different strains when multiple strains of the same species are infecting a single animal. Therefore, the new ST of *L. interrogans* identified in this study needs to be confirmed in an isolate. Although sample-to-sample contamination cannot be entirely excluded, this is unlikely because double peaks were not consistently observed across all loci but rather appeared only in specific genes, and because negative controls consistently remained negative. Moreover, the presence of alleles corresponding to different species in the same specimen strongly suggests genuine co-infection rather than laboratory contamination.

There are several limitations to this study. First, we focused exclusively on rodents and did not include other potential reservoir animals that may contribute to human infections. Second, the novel STs of *L. interrogans* and *L. kirschneri* identified in this study have not yet been detected in human patients, and therefore, their zoonotic potential and transmission pathways remain to be determined. Finally, our conclusions regarding the reservoir role of rodents are based solely on the detection of leptospiral DNA in kidney tissues, and isolation of viable organisms from urine would be valuable to confirm active shedding, although such attempts are technically challenging because obtaining uncontaminated urine from small wild rodents is difficult.

In conclusion, this study demonstrates the genetic diversity of *Leptospira* spp. in rodent reservoirs in Sri Lanka, highlighting the complexity of *Leptospira* ecology and the disease transmission to humans. In addition, the high frequency of mixed infections highlights the diagnostic and clinical challenges associated with coinfections. There are still several genotypes detected only in human patients, such as ST323 in this and previous studies [25,26], and continued surveillance of rodents and other reservoir animals and genetic analysis of *Leptospira* isolates are needed to better understand transmission pathways, develop effective strategies for controlling leptospirosis and improve public health interventions in Sri Lanka.

## Supplementary material

10.1099/jmm.0.002133 Uncited Supplementary Material 1.

## Abbreviations

*Cytb*: cytochrome b
*flaB*: flagellin *B*
MLST: multilocus sequencing typing
MST: minimum spanning tree
STs: sequence types
WGS: whole-genome sequencing
