# Supplementary material for: Molecular epidemiology of rodent-borne Leptospira spp. in Sri Lanka: identification of novel sequence types and previously unrecognized reservoir animals
Source: J Med Microbiol. 2026 Mar 6;75(3):002133. doi: 10.1099/jmm.0.002133 (PMC12967096; doi:10.1099/jmm.0.002133)
Supplement: Uncited Supplementary Material 1. [file jmm-75-02133-s001.pdf]

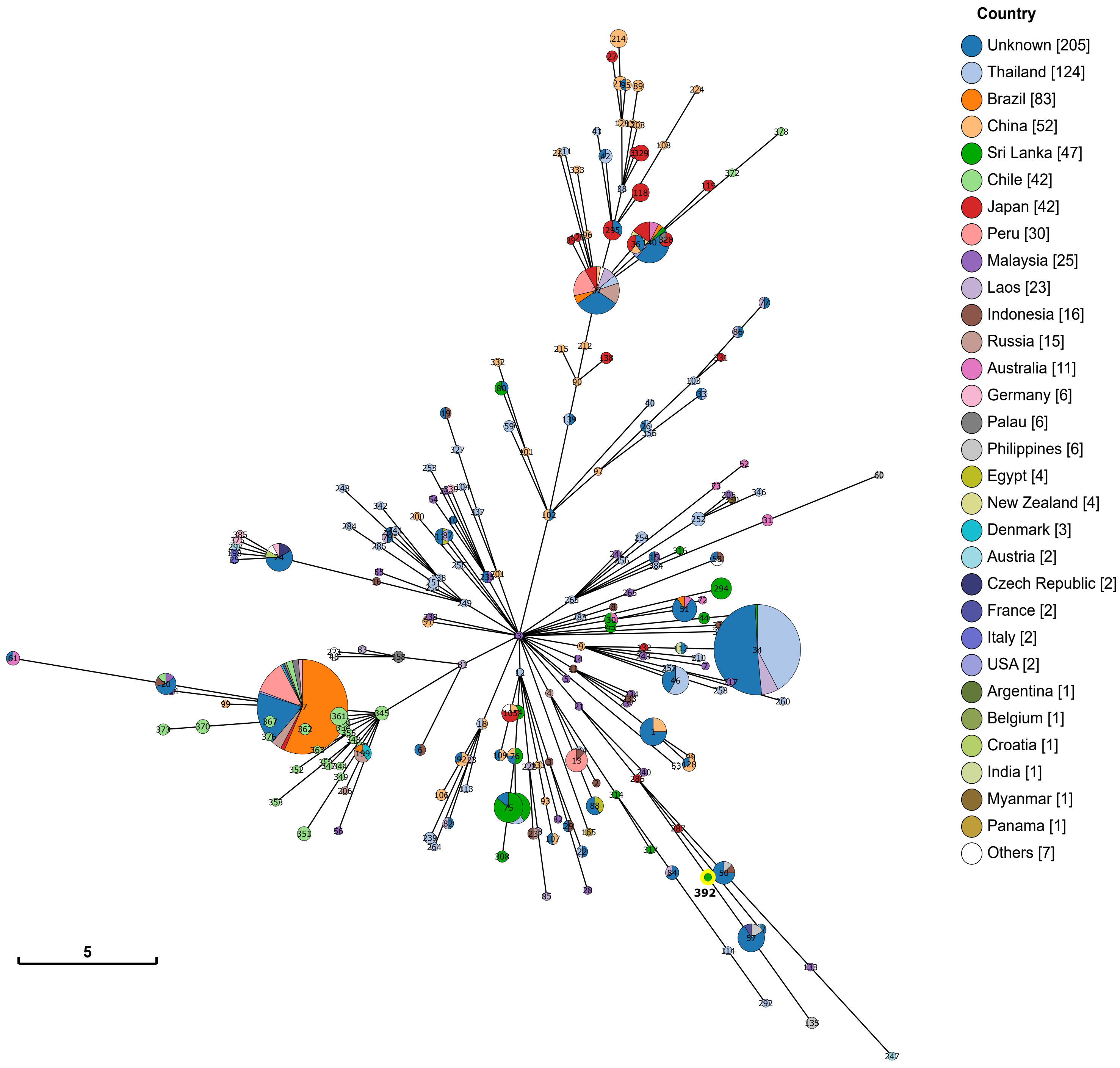

Supplementary Fig. 1. Minimum spanning tree of *Leptospira interrogans* sequence types (STs) identified in this study and those deposited in the *Leptospira* PubMLST database.

Each circle represents a ST, and the numbers indicate ST designations. Circle sizes are proportional to the number of strains belonging each ST. Yellow-highlighted circles indicate the new ST identified in this study. Node colors represent the country of origin of each strain. The MST was constructed based on allelic profiles from the seven housekeeping genes of MLST scheme 1.

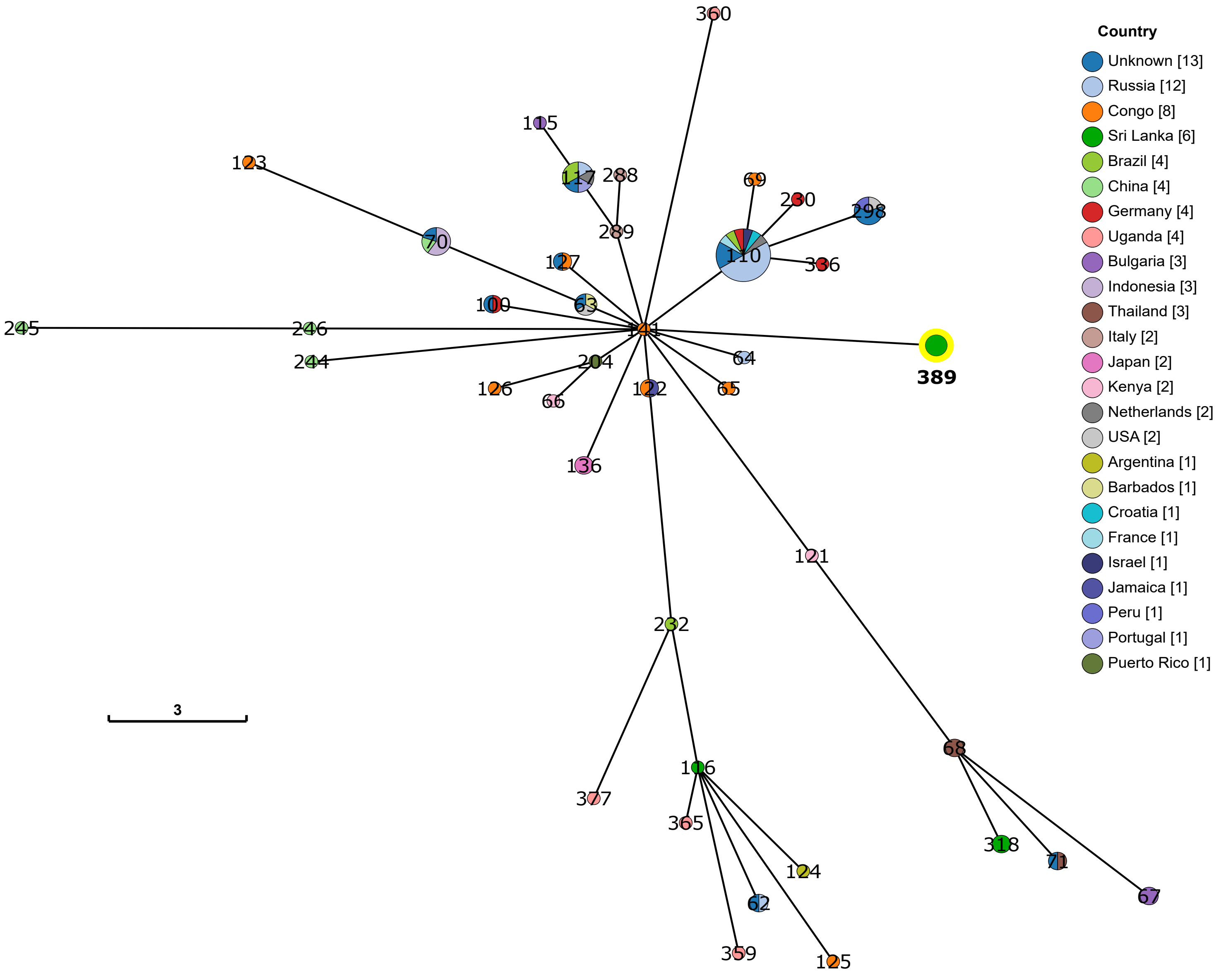

Supplementary Fig. 2. Minimum spanning tree of *Leptospira kirschneri* sequence types (STs) identified in this study and those deposited in the *Leptospira* PubMLST database.

Each circle represents a ST, and the numbers indicate ST designations. Circle sizes are proportional to the number of strains belonging each ST. Yellow-highlighted circles indicate the new ST identified in this study. Node colors represent the country of origin of each strain. The MST was constructed based on allelic profiles from the seven housekeeping genes of MLST scheme 1.
